# Anti-Diabetic Effects of *Amygdalus Lycioides *Spach in Streptozocin-Induced Diabetic Rats

**Published:** 2018

**Authors:** Leila Moezi, Seyede Samira Arshadi, Tahere Motazedian, Seyed Hassan Seradj, Farzaneh Dehghani

**Affiliations:** a *Department of Pharmacology, School of Medicine, Shiraz University of Medical Sciences, Shiraz, Iran.*; b *Department of Pharmacognosy, School of Pharmacy, Shiraz University of Medical Sciences, Shiraz, Iran. *; c *The Persian Gulf Tropical Medicine Research Center, Bushehr University of Medical Sciences, Bushehr, Iran. *; d *Histomorphometry and Stereology Research Center, School of Medicine, Shiraz University of Medical Sciences, Shiraz, Iran.*

**Keywords:** *Amygdalus lycioides*, Streptozocin, Diabetes, Pancreas, Stereology, β-cell, Rat

## Abstract

Diabetes mellitus is a group of metabolic disorders characterized by elevated blood sugar and abnormalities in insulin secretion and action. There are many anti-diabetic plants, which might supply useful sources for developing new medicines that can be used in treatment of diabetes mellitus. The primary objective of the present investigation is to evaluate the anti-diabetic properties of the aerial parts of *Amygdalus lycioides* in streptozocin-induced diabetic rats. Sixty rats were divided into 6 groups: streptozocin-induced diabetic control, insulin-treated diabetic group, and four *Amygdalus lycioides*-treated diabetic groups (125, 250, 500, and 1000 mg/kg/day). After 2 weeks of plant extract administration, the effects of extracts on blood glucose, body weight, BUN, creatinine, total cholesterol, LDL, HDL, triglyceride, total protein, Na, K, and plasma enzymes (aspartate aminotransferase, alanine aminotransferase and alkaline phosphatase) were analyzed. The pancreas of rats was also stained for stereological studies. Phytochemical evaluation of this extract showed the presence of flavonoids and tannins compounds. Glucose serum levels and glucose tolerance test showed a decrease in treatment with *Amygdalus lycioides *(1000 mg/kg). Serum total cholesterol, LDL, triglyceride, creatinine and alkaline phosphatase levels were decreased significantly by the extract but aspartate aminotransferase found to be increased after treatment. The total number and numerical density of beta cells increased in the *Amygdalus lycioides *group (1000 mg/kg). It seems that *Amygdalus lycioides *may act as a potential drug to treat diabetes and its complications. However, more investigations should be done to more clarify these results.

## Introduction

Diabetes Mellitus (DM) is a complex metabolic endocrine disorder that affecting the metabolism of carbohydrates, proteins and lipids (1, 2). It is a condition in which the amount of glucose increases in the blood, because organs *e.g.* adipose tissues and liver does not properly utilize the glucose (3). The prevalence of diabetes among adults within the 20–79 year range was estimated to be 6.4% in 2010, affecting 285 million people worldwide. The prevalence is expected to rise to 7.7% and 439 million adults by 2030 (4).

Diabetes is associated with a number of chronic complications and co-morbidities. The most prevalent and well known complications include retinopathy, nephropathy, and peripheral neuropathy (5). Insulin has the most important role to control the level of blood glucose by releasing from the pancreas after meal. Although insulin therapy, oral hypoglycemic agents, restricted diet and exercise either singly or in combination are the most effective regimen of therapy for the diabetic patients today, in a large number of cases treating with traditional medicine in the form of plant extracts have been reported to give good results . There are many anti-diabetic plants, which might supply useful sources for developing new medicines that can be used in treatment of diabetes mellitus. Ethno-botanical information indicates that more than 800 plants are used as traditional remedies for the treatment of diabetes (6), but many plants do not have a scientific scrutiny. Some of these plants have been pharmacologically tested and shown to be of some values in diabetes (7-10).

*Amygdalus lycioides *Spach, a plant belonging to the rosaceae family is an endemic species extending into South Anatolia (11). Almond species grow under subtropical Mediterranean climate, with mild wet winters and warm and dry summers. They originated from central Asia and represent divergent evolution under xerophytic environments. Related Prunus species are found growing wild from eastern China to mountainous areas and deserts of Western China, Kurdistan, Turkistan, Afghanistan, and Iran (12).

In Iranian folk medicine *Amygdalus lycioides *is often known as “Badam Talkhkuhi” and has been used as anti-inflammatory and anti-microbial herbal remedy since ancient times (13). The aerial parts and roots of *Amygdalus lycioides *are also used in the treatment of diabetes in Iranian ancient medicine (14). In spite of the widespread use of *Amygdalus lycioides*, there is no scientific investigation on its anti-diabetic effect. This study pointed out to find if hydroalchoholic extract of *Amygdalus lycioides *can have any effect on biochemical parameters of diabetic rats including plasma glucose, BUN, creatinine, total cholesterol, TG, LDL, HDL, total protein, Na, K and plasma enzymes which are indicators of liver function (aspartate aminotransferase, alanine aminotransferase and alkaline phosphatase). We also investigated its potential ability to regenerate pancreas using stereology method.

## Experimental

*Chemicals*

The following drugs were used throughout the study: NPH insulin was purchased from Wolfratshausen (Germany). Streptozocin was purchased from Sigma (USA) and thiopental was purchased from Sandoz (USA). Streptozocin was dissolved in 0.1 M citrate buffer (pH 4.5). NPH insulin, thiopental, and streptozocin (60 mg/kg) were administered intraperitoneally, while the extract was administered orally by gavage. 

*Animals*

The experimental animals were male Sprague–Dawley rats (200–300 g body weight) procured from Shiraz University of Medical Sciences. They were maintained under standard conditions (temperature 22 ± 2 °C, relative humidity of 60 ± 5% and 12 h light/dark cycle). They had free access to standard pellet diet and water ad libitum. The Institutional Animal Ethics Committee of Shiraz University of Medical Sciences approved the experimental protocol. All the animals received animal care according to the criteria outlined in the «Guide for the Care and Use of Laboratory Animals». 

*Plant Material*

*Amygdalus lycioides *Spach branches were collected near Shiraz (Bavanat), Iran, in June 2013. It was identified by a specialist botanist. A voucher specimen (V. No. 765) was deposited in the Herbarium of Shiraz University of Medical Science, Iran. The air-dried plant material was stored in dark conditions and then blended to make soft powder.

*Extraction*

The powder (1000 g) of *Amygdalus lycioides *Spach were pre-treated with ethanol 50% (w/v) for 48 h in room temperature. The extract was separated by filtration and solvent was evaporated to dryness under vacuum to 55 °C, yielding a dried residue (3.8 g). Ten g of dried residue was solved in 100 mL water to make the extract.

*Preliminary phytochemical screening of Amygdalus lycioides*

The preliminary phytochemical analysis including ﬂavonoids, tannins and alkaloids determination tests was carried out for the extract of *Amygdalus lycioides*, using standard phytochemical methods (15).

*Standardization of the plant according to total flavonoids contents*

In order to make the standardization of the plant according to total flavonoids contents, we determined total flavonoids in the plant extract. Aluminum chloride method was used for the determination of the total flavonoid content of the sample extracts (16). Aliquots of extract solutions were taken and made up the volume 3 mL with methanol. Then 0.1 mL AlCl3 (10%), 0.1 mL Na-K tartrate and 2.8 mL distilled water were added sequentially. The test solution was vigorously shaken. Absorbance at 415 nm was recorded after 30 min of incubation. A standard calibration plot was generated at 415 nm using known concentrations of quercetin. The concentrations of flavonoids in the test samples were calculated from the calibration plot and expressed as mg quercetin equivalent/g of sample.

*Diabetes Induction*

After overnight fasting, diabetes was induced by intraperitoneal injection of streptozocin dissolved in 0.1 M cold sodium citrate buffer (pH 4.5) at a dose of 60 mg/kg which can induce type I diabetes mellitus (17). Blood samples were taken from tail vein before and after the start of experiment. Blood glucose was determined using a glucose monitoring (accu-check, Roche, USA). After 4 days, the time for the development of diabetes, the rats with moderate diabetes having hyperglycemia (blood glucose range of above 250 mg/dL) were considered as diabetic rats and used for the future experiments.

*Experimental groups*

Animals were randomly divided into six groups and each group contained 10 diabetic rats. Group I served as solvent-treated diabetic control group and group II served as insulin group that received insulin NPH (3 unit) subcutaneously each day. In groups III, IV, V and VI, plant extract (125, 250, 500 and 1000 mg/kg, respectively) were administrated once daily orally along with normal food.

Treatment of experimental animals with plant extracts or insulin was initiated 4 days post streptozocin injection and drug administration was carried out once daily for 14 days. Food and water were made freely available. The initial and ﬁnal body weights were measured. Blood samples for glucose determination were obtained from the tail tip of 12 h fasted rats on day 0 (before streptozocin administration) and 4, 11 and 18 days after streptozocin injection. On the 18^th^ day post streptozocin injection, the animals were fasted for 12 h. After fasting blood glucose determination from the tail tip of rats, glucose (2 g/kg) was fed to rats. Blood was withdrawn from the tail vein at 120 min and glucose levels were estimated (oral glucose tolerance test: OGTT). After that rats were anaesthetized using thiopental and sacriﬁced by decapitation. Blood was collected from the heart in a dry tube and allowed to coagulate at ambient temperature for 30 min. Serum was separated by centrifugation at 2000 rpm for 10 min, then aspartate transaminase (AST), alanine aminotransferase (ALT), alkaline phosphatase (ALP), cholesterol, triglycerides, HDL, LDL, Na, K, creatinine, urea, and total protein levels in different groups of rats were determined.

*Stereological study*

**Figure 1:**
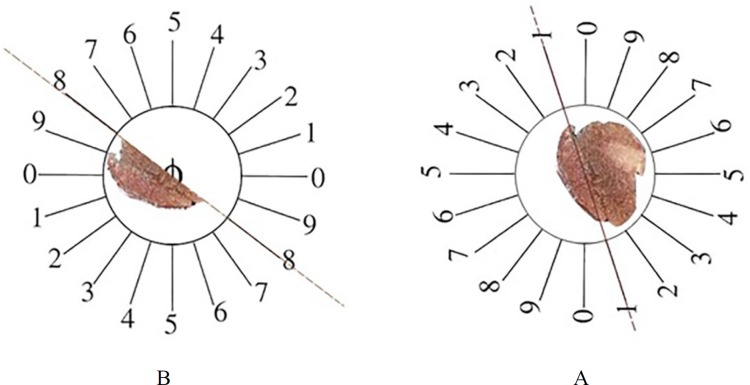
The orientator method to obtain the isotropic uniform random (IUR) sections. (A) The whole pancreas was placed on the one circle and a random number choose randomly. Then the pancreas was cut at that direction (here, it was 1). (B) Then, the other part of pancreas was located in the other circle and the second cut at a random number (here, it was 8).

At the end of experiment, the animals were dissected and the pancreas were removed and weighed. Primary volume V (primary) of the pancreas was measured using the immersion method (18). In some cases, shrinkage could occur after fixation and tissue preparation. These factors may have effe­cts on the quantitative estimation; therefore, it seems necessary to measure tissue shrinkage (19, 20). Orientator method to obtain isotropic uniform random (IUR) sections of pancreas was used for estimation of pancreas shrinkage. In this method, the pancreas was located at the center of a circle and a number was selected randomly, then the pancreas was sectioned. Each portion of pancreas was located on the other circle ­­and a new number was selected, and then the pancreas was cut into slabs. Another portion of pancreas was located on the same circle and sectioned into slabs in a new axis. In this procedure we obtained about 8-12 slabs of pancreas. For estimation of pancreas shrinkage, two circles of tissue were punched by trocar and measured pre-fixing radius (r before) and post-fixing radius (r after) (Figure 1A).

Volume shrinkage of pancreas was estimated by using the following formula:

Volume _shrinkage_ = 1 – (r _after2_ /r _before2_) ^1.5^

After volume of shrinkage was estimated, the tissue was fixed by buffer formaldehyde, embedded by paraffin, sectioned and stained by modified aldehyde fuchsia (21). The 5 µm thin serial sections and 20 µm thick pair serial sections were cut in order to estimate the volume and number of islets and β-cell, respectively.

The final volume was estimated by following formula:

Final volume: V_primary _× (1 - volume_shrinkage_)

*Volume Density of the Pancreatic Islets*

The volume density of islet was estimated by using the point-counting method. In this method, the 5 µm microscopic slide of 8 to 10 sections from each tissue was analyzed using a video microscopy system (E-200, Nikon, Tokyo, Japan) linked to a video camera, and a computer, with a flat monitor. On each section, 6 to 8 fields were randomly selected by systematic random manner. 

**Figure 2.**
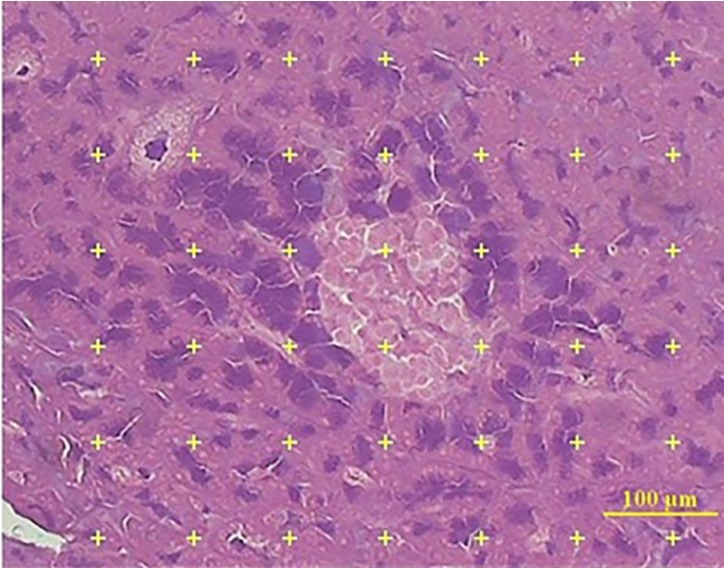
Estimation of Langerhans islet volume using point counting method. Eight to twelve × sections from each pancreas were prepared by formula. A Pointing test system was overlaid on the image of the islet of pancreas (4X, aldehyde fushin staining

The pointing test grids were superimposed on the images and viewed on the monitor by means of the stereology software and analyzed using a microscope (Figure 2). This software was designed by our research center (Stereological Research Laboratory, Shiraz University of Medical Sciences, Shiraz, Iran).

The number of the points hitting the reference space (the pancreas) and the islets estimated the volume density (Vv) of the pancreatic islet was estimated using the following formula:

V_v_ = P_islet_ / P_reference_

Where, P (islet) and P (reference) were the number of the points falling on the islet’s profile and on the reference space, respectively. The following formula was used to estimate the final islet volume: 

V_islets_ = V_v_ × V _final volume_

by multiplying the volume density of the islet by the final volume of the pancreas.

*Number of β- cells*

**Figure 3:**
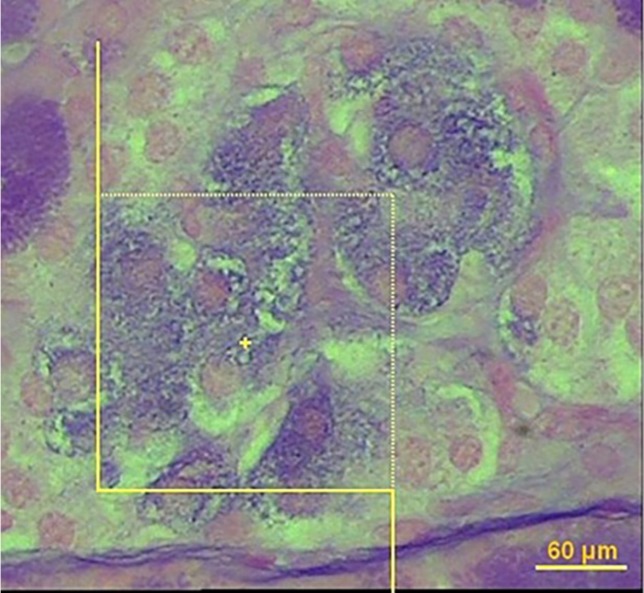
Estimation of the number of β-cell using the optical disector. The nucleoli profiles of the β-cell were counted, in solvent-treated diabetic group (A), *Amygdalus lycioides *(1000 mg/kg) extract-treated group (B), and insulin-treated diabetic group (C). The nucleoli were counted only if they were inside or partially inside the sampling frame and none of their parts touched the exclusion lines of the frame (40X, aldehyde fushin staining

In order to estimate the number of β-cell, it is necessary to obtain the numerical density of the β-cells using the optical disector method (Figure 3). In this method, the 20 µm microscopic slide was analyzed using a video microscopy system (E-200, Nikon, Tokyo, Japan) linked to a video camera, a computer, a flat monitor, and a microcator (MT-12, Heidenhain Traunreut, Germany). The height of disector or “h” is distance between two pair sections and the first 5 μm of the section thickness was ignored to avoid biased counting (guard zone). To determine the volume density of β-cell, 8-10 microscopic fields were selected through systematic uniform random sampling. To estimate the β-cell, we used about 90 systematic frames applied on pancreatic islands. Nuclei of β-cells were considered as the whole cell. The cells whose nuclei were completely or partly inside the counting frame or touched the upper and right lines were counted (ΣQ). Only the nuclei did not appear at the beginning of the disector height and appeared in the following optical scan of the disector height were counted. 

The numerical density (NV) was estimated using the optical disector method and the following formula:

Nv = ∑Q/h. a/f. ∑p

Where: 

∑Q: is the number of β-cell

h: is the height of optical dissector

a/f: area of the test frame

∑p: is the number of test frame on the islet profile

To estimate the number of the β-cell, the following formula was used:

N _β-cell_ = N_v_. V _islet_

*Statistical analysis*

The results were analyzed using One-Way analysis of variance (ANOVA) followed by Tukey post-hoc test. *P* < 0.05 was considered statistically significant. 

## Results

*Preliminary phytochemical analysis*

The preliminary phytochemical evaluation of the extract showed the presence of ﬂavonoids and tannins compounds.

*Standardization*

**Figure 4:**
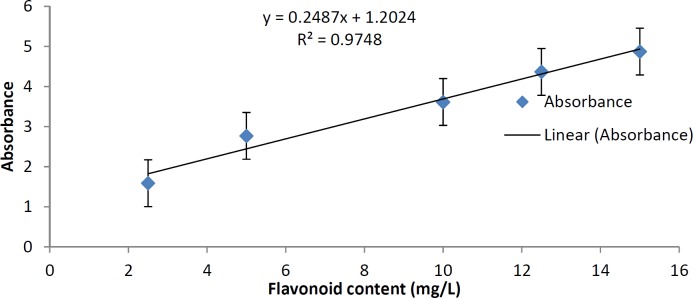
Standard plot of quercetin

The concentration of flavonoid in our extract was calculated using the standard plot of quercetin (y = 0.2487x + 1.2024, R² = 0.9748). The results showed that our extract according to its absorbance in 415 nm, has 11.24 mg quercetin equivalent/g of extract (Figure 4).

*The effects of the Amygdalus lycioides extract on body weight of animals*

The effects of the *Amygdalus lycioides* extract on the body weight of diabetic rats are shown in Table 1. During the first week of observation of the diabetic rats after drugs administration, there was no significant alteration of weight of animals. When we measured the weight of animals two weeks after the administration of drugs, there was a significant weight gains of insulin-treated diabetic rats compared to the diabetic rats which received solvent (*P *< 0.01). The diabetic rats treated with the extract, did not show any changes in their weights during 2 weeks of treatment (*P *> 0.05).

*The effects of the extract on blood sugar*

After 2 weeks of treatment with the extracts, the fasting glycemic level of *Amygdalus lycioides* extract (1000 mg/kg)-treated diabetic rats dropped significantly (*P *< 0.001). Insulin-treated diabetic rats also showed significant decrease in fasting blood sugar levels compared to diabetic rats one and two weeks after treatment (*P *< 0.001 and *P *< 0.01, respectively) (Table 2).

The changes in serum glucose of rats, 120 min after oral administration of glucose has been shown in Table 2. The extract at dose of 1000 (mg/kg) produced a signiﬁcant attenuation in OGTT level when compared to the diabetic un-treated rats (*P *< 0.01). Insulin also caused signiﬁcant attenuation in serum glucose of rats, 120 min after oral administration of glucose when compared to the diabetic un-treated rats (*P *< 0.01).

**Table 1:** The effect of treatment with different concentrations of *Amygdalus lycioides* extract on body weight in streptozocin-induced diabetic rats

**Group**	**Initial weight (g)**	**Weight 11 days after streptozocin (g)**	**Weight 18 days after streptozocin (g)**
Solvent	234.5 ± 9.608	220.166 ± 9.683	190.333 ± 9.898
*Amygdalus lycioides* (125 mg/kg)	233.5 ± 9.854	208.833 ± 11.028	171.833 ± 11.92
*Amygdalus lycioides* (250 mg/kg)	241.666 ± 8.811	201.333 ± 10.048	166.333 ± 10.265
*Amygdalus lycioides* (500 mg/kg)	253 ± 11.958	215.166 ± 9.481	178 ± 8.884
*Amygdalus lycioides* (1000 mg/kg)	253 ± 10.49	229.6 ± 9.83	184.83 ± 4.06
*Insulin NPH (3 IU)*	248.5 ± 7.561	254.166 ± 6.337	249 ± 9.615**

Data are expressed as mean ± SEM of body weight in each group.

** *P *< 0.01, compared with corresponding solvent-treated diabetic group.

**Table 2.** The effect of treatment with different concentrations of *Amygdalus lycioides* extract on serum fasting blood glucose (FBS) and oral glucose tolerance test (OGTT) in streptozocin-induced diabetic rats

**Groups**	**Initial FBS** **(mg/dL)**	**FBS 4 days after streptozocin (mg/dL)**	**FBS 11 days after streptozocin (mg/dL)**	**FBS 18 days after streptozocin (mg/dL)**	**OGTT** **(mg/dL)**
Solvent	76.16 ± 3.80	497.33 ± 28.90	367 ± 58.82	454 ± 6.44	633.5 ± 4.78
*Amygdalus lycioides* (125 mg/kg)	77 ± 3.06	495.66 ± 29.53	387.5 ± 9.25	423 ± 13.69	579 ± 16.35
*Amygdalus lycioides* (250 mg/kg)	80.33 ± 3.01	461.33 ± 33.96	339 ± 35.19	353 ± 65.93	538.66 ± 51.84
*Amygdalus lycioides* (500 mg/kg)	75.83 ± 3.62	506.66 ± 26.29	320.66 ± 47.49	362 ± 60.03	537.83 ± 41.4
*Amygdalus lycioides* (1000 mg/kg)	82.5 ± 5.03	437.33 ± 22.11	244 ± 35.28	147 ± 38.59***	375.83 ± 77.67**
*Insulin NPH (3 IU)*	75.66 ± 2.55	505.66 ± 36.80	93.5 ± 20.05***	207.16 ± 75.23**	329.83 ± 73.19**

Data are expressed as mean ± SEM of data in each group.

** *P *< 0.01 and

*** *P *< 0.001, compared with corresponding solvent-treated diabetic group.

**Table 3:** The effect of treatment with different concentrations of *Amygdalus lycioides* extract on serum total cholesterol, LDL, HDL, and triglyceride in streptozocin-induced diabetic rats

**Groups**	**Total cholestrol (mg/dL)**	**LDL (mg/dL)**	**HDL (mg/dL)**	**Triglyceride (mg/dL)**
Solvent	54.83 ± 3.13	19.2 ± 2.47	20.33 ± 1.08	89.66 ± 9.66
*Amygdalus lycioides* (125 mg/kg)	46.16 ± 6.24	13.5 ± 2.92	16.5 ± 1.94	80.66 ± 10.58
*Amygdalus lycioides* (250 mg/kg)	42.83 ± 7.02	12.16 ± 4.77	15.83 ± 1.5	75 ± 5.83
*Amygdalus lycioides* (500 mg/kg)	35.33 ± 6.3*	4.2 ± 0.73**	14.16 ± 2.12	64.83 ± 2.63*
*Amygdalus lycioides* (1000 mg/kg)	28.16 ± 1.01**	6.66 ± 1.72*	16.5 ± 1.87	26.66 ± 3.07***
*Insulin NPH (3 IU)*	53.83 ± 3.17	18.5 ± 1.66	21.83 ± 1.51	67.5 ± 2.04

Data are expressed as mean ± SEM of data in each group.

* *P* < 0.05,

** *P *< 0.01 and

*** *P *< 0.001, compared with corresponding solvent-treated diabetic group.

**Table 4:** The effect of treatment with different concentrations of *Amygdalus lycioides* extract on serum AST, ALT, ALP, total protein, creatinine, BUN, Na and K in streptozocin-induced diabetic rats.

**Groups**	**AST (U/dL)**	**ALT (U/dL)**	**ALP (U/dL)**	**Total Protein (g/dL)**	**Creatinine (mg/dL)**	**BUN (mg/dL)**	**Na (mg/dL)**	**K (mg/dL)**
Solvent	276.5 ± 82.01	408.66 ± 206.64	2247.16 ± 130.96	7.38 ± 0.22	0.65 ± 0.05	60.16 ± 3.67	141.66 ± 1.02	4.76 ± 0.6
*Amygdalus lycioides* (125 mg/kg)	428.5 ± 46.12	279.83 ± 42.47	2676.83 ± 239.38	7.31 ± 0.43	0.68 ± 0.04	63.83 ± 7.5	143.83 ± 0.6	6.35 ± 0.82
*Amygdalus lycioides* (250 mg/kg)	462.33 ± 98.28	241.5 ± 51.46	2023.16 ± 260.88	7.15 ± 0.47	0.65 ± 0.07	72.33 ± 6.74	143 ± 0.57	6.85 ± 0.66
*Amygdalus lycioides* (500 mg/kg)	753.66 ± 230.06	251 ± 74.78	1708 ± 234.54	6.11 ± 0.24	0.45 ± 0.05*	64.83 ± 7.11	143.5 ± 0.71	5.68 ± 0.86
*Amygdalus lycioides* (1000 mg/kg)	977.33 ± 201.08**	357.33 ± 42.49	1005.83 ± 61.37***	5.83 ± 0.09	0.61 ± 0.03	59.66 ± 1.05	141.66 ± 0.66	5.46 ± 0.42
Insulin NPH (3 IU)	225.5 ± 22.56	46.66 ± 2.61*	354.5 ± 18.26***	6.73 ± 0.17	0.46 ± 0.02	19.16 ± 1.85***	141.16 ± 1.04	3.8 ± 0.12

Data are expressed as mean ± SEM of data in each group.

* *P* < 0.05 and

*** *P* < 0.001, compared with corresponding solvent-treated diabetic group.

**Figure 5.**
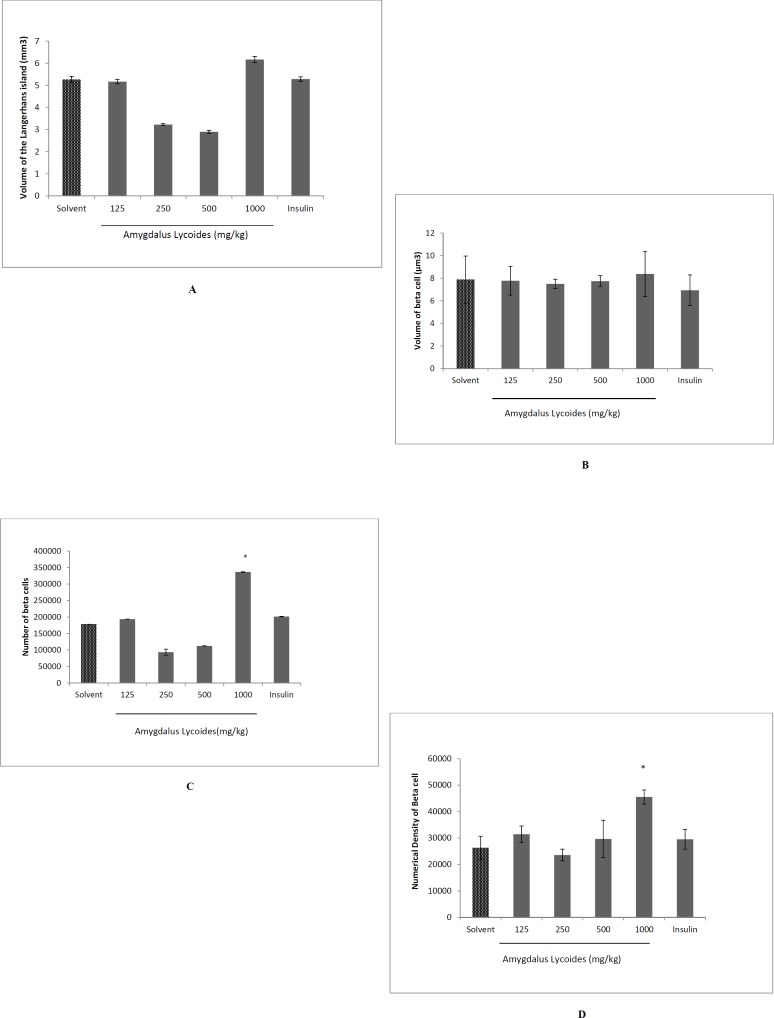
The effect of treatment with different concentrations of *Amygdalus lycioides* extract on volume of the Langerhans island (A), volume of beta cells (B), total number of beta cells (C), and numerical density of beta cells (D) on pancreas of streptozocin-induced diabetic rats. Each group contained 10 diabetic rats. Data are expressed as mean ± SEM in each group. *P < 0.05 compared with solvent-treated diabetic group

*The effects of the extract on blood lipids*

Extract-treated rats (500 and 1000 mg/kg), showed a signiﬁcant decrease in total cholesterol (*P *< 0.05 and *P *< 0.01, respectively), LDL (*P *< 0.01 and *P *< 0.05, respectively) and triglyceride levels (*P *< 0.05 and *P *< 0.001, respectively), but there was no significant difference between HDL levels of serums in diabetic rats treated by the extract and the solvent (*P *> 0.05) (Table 3).

*The effects of the extract on other biochemical levels*

Serum AST levels were increased in extract-treated (1000 mg/kg) diabetic rats (*P *< 0.01) while ALP levels were decreased in extract-treated (1000 mg/kg) and insulin-treated diabetic rats (*P *< 0.001). ALT and total protein levels remained unchanged in all extract-treated groups (*P *> 0.05) (Table 4).

Serum creatinine levels of the extract group (500 mg/kg) decreased significantly (*P *< 0.05), while BUN serum levels were decreased only in NPH-treated diabetic animals (*P *< 0.001). Besides that Na+ and K+ levels were unchanged in all treated groups (*P *> 0.05) (Table 4).

*Volume of the Langerhans island and beta cells*

There was not any significant difference in the total volume of Langerhans island and volume of beta cells between groups (*P *> 0.05) (Figures 5A and 5B).

*The total number and numerical density of beta cells*

The total number of beta cells increased in the extract group (1000 mg/kg) compared to diabetic control group and insulin group (*P *< 0.05). Furthermore, the numerical density of beta cells increased in the extract group (1000 mg/kg) in comparison to diabetic normal control and insulin group (*P *< 0.05) (Figures 5C and 5D).

## Discussion

The present study discusses about anti-diabetic potential effect of hydroalchoholic extract of *Amygdalus Lycioides *Spach. To our knowledge this is the first study evaluating the effect of a hydroalcoholic *Amygdalus lycioides* extract on diabetic rats.

Streptozocin has been used as a diabetogenic agent and low doses of it were used to induce diabetes mellitus in animals (22). It has been demonstrated that streptozocin, depending on its dose, extensively reduces beta cells mass and destroys pancreatic islet volume (23, 24). Additionally, the prolonged toxicity of hyperglycemia exhausts the remaining viable beta cells and worsens diabetes (25). In the present study, streptozocin administration induced pronounced increase in the concentration of blood glucose. A significant hyperglycemia was attained within 4 days after streptozocin administration and remained for at least 14 days.

Plant derived anti-diabetic agents are gaining popularity day by day around the world for their effective anti-diabetic activity and minimal side effects (26). In this study, we indicated that *Amygdalus lycioides *(1000 mg/kg) caused decrease in plasma fasting glucose and glucose tolerance test.

There are many bioactive compounds in plants that have hypoglycemic effect such as flavonoids, phenolic compound, terpenoids and alkaloids (27). In this study we demonstrated the presence of ﬂavonoids in the aerial parts of *Amygdalus lycioides*. Previous studies also reported that *Amygdalus lycioides *contains flavonoids including quercetin 3-O-rhamnoside, luteolin 7-O-rhamnoside, isorhamnetin 3-O-rutinoside, kaempferol 3-O-rhamnoside, apigenin, and naringenin (14), so its hypoglycemic effect may be refer to these bioactive components. Investigations showed that flavonoids can reduce the level of plasma glucose. Flavonoids such as quercetin can inhibit glucose absorption in intestine by specific acting on GLUT2 transporter (28). 

The other possible mechanism of activity of *Amygdalus lycioides* leaves extract may be stimulating the insulin secretion and regeneration of the β-cells of the pancreas or increasing cellularity of the islet tissue and regeneration of the granules in the β-cells. This hypothesis has been confirmed by the stereological studies of the pancreas which showed protection of the pancreas from the toxic effect of streptozocin. We indicated that the total number and numerical density of beta cells increased in the *Amygdalus lycioides *group (1000 mg/kg) but the volume of Langerhans Island remained unchanged. We suppose that *Amygdalus lycioides* in this dose stimulates stem cells and differentiates these cells to β-cells. The extra-cellular matrix is an essential component of stem cell. The proliferation, differentiation, and behavior of stem cells depend on extra-cellular matrix (29). Extracellular matrix of stem cells has small amount of fibers. It seems that *Amygdalus lycioides* stimulates β-cells proliferation but reduces extra-cellular matrix. According to this hypothesis *Amygdalus lycioides* did not affect volume of islands. 

Several studies left little doubt that inflammatory pathways are critical in the mechanisms underlying insulin resistance and type 2 diabetes (30); where even minimal disturbances in glucose tolerance are associated with a chronic, generalized inflammatory reaction that links components of the metabolic syndrome and contributes to the development of diabetic complications. The circulating markers of inflammation (*e.g.*, TNF-α and IL-6) and acute-phase reactants (*e.g.*, CRP) are considered strong predictors of the development of type 2 diabetes and the possible associated cardiovascular complications (31). Anti-inflammatory agents protect pancreatic beta-cells by attenuating inflammatory responses in diabetes treatment (32).

It has been also demonstrated that the antioxidant defense against reactive oxygen species protects β-cells against loss, and exhibit antidiabetic property (33). Previous studies showed that flavonoids from *Amygdalus lycioides* are known to exhibit strong antioxidant scavenging activity (14). Potential antioxidant (14) and anti-inflammatory activity (34) of flavonoids from *Amygdalus lycioides *may prevent from progressive STZ induced damages of beta cells.

In this study, *Amygdalus lycioides* extract did not induce any significant changes in body weight of all groups. The first point of this result is that *Amygdalus lycioides* could not prevent the weight reduction induces by diabetes (35) and the second point is that *Amygdalus lycioides* does not have a weight gain side effect such as insulin and sulfonylurea.

Diabetes mellitus is associated with a dyslipidaemic profile. This lipid dysregulation makes an important contribution to the elevated cardiovascular risk associated with diabetes (36). In the current study we demonstrated that extract-treated rats (500 and 1000 mg/kg), showed a signiﬁcant decrease in total cholesterol, LDL, and triglyceride levels, but there was no significant difference between HDL levels of serums in diabetic rats treated by the extract. Therefore anti-lipidemic effect of the extract could be helpful in diabetes. 

Diabetic nephropathy is also a high risk factor for vascular disease (37). Diabetic nephropathy is structural abnormalities revealing hypertrophy of both glomerular and tubular elements. Eventual losses of renal functions are signs of diabetic nephropathy (37, 38). In the present study, serum creatinine levels of extract group (500 mg/kg) decreased significantly, while BUN serum levels remained unchanged in the all extract groups. Thus the extract not only did not increase the kidney related serum factors but also improved creatinine serum levels. 

Treatment of diabetes might require the use of these extract for a longer time. But the drugs were examined here only for 14 days. Longer duration studies are necessary to find out whether prolonged treatment with *Amygdalus lycioides* would have different results.

In conclusion, *Amygdalus lycioides *may act as a potential drug to decrease glucose and lipids in treating diabetes and its complications. Anti-diabetic action of *Amygdalus lycioides* may, at least partly, be mediated through flavonoids and pancreas regeneration. 

## References

[1] Dehwah MAS, Shuang Z, Huang QY (2008). The association between ACE gene I/D polymorphism and type 2 diabetes in Han Chinese in Hubei. Int. J. Osteoporos. Metab. Disord.

[2] Alhazza I, Bashandy SA (2007). Hypoglycemic, hypolipidemic, antioxidant and male sexual improvement potentials of olive oil in alloxan treated rats. J. Pharmacol. Toxicol.

[3] Kumar V, Anwar F, Ahmed D, Verma A, Ahmed A, Damanhouri ZA, Mishra V, Ramteke PW, Bhatt PC, Mujeeb M (2014). Paederia foetida Linn leaf extract: An antihyperlipidemic antihyperglycaemic and antioxidant activity. BMC Complement. Altern. Med.

[4] Shaw JE, Sicree RA, Zimmet PZ (2010). Global estimates of the prevalence of diabetes for 2010 and 2030. DiabetesRes. Clin. Pract.

[5] Mihanatzidou E, Aud M, Kerlew R (2014). The link between diabetes mellitus and sensorineural hearing loss: A summary of the evidence. Canadian Hearing Report.

[6] Pushparaj PN, Low HK, Manikandan J, Tan BK, Tan CH (2007). Anti-diabetic effects of Cichorium intybus in streptozotocin-induced diabetic rats. J. Ethnopharmacol.

[7] Nmila R, Gross R, Rchid H, Roye M, Manteghetti M, Petit P, Tijane M, Ribes G, Sauvaire Y (2000). Insulinotropic effect of Citrullus colocynthis fruit extracts. Planta Med.

[8] Sauvaire Y, Petit P, Broca C, Manteghetti M, Baissac Y, Fernandez-Alvarez J, Gross R, Roye M, Leconte A, Gomis R (1998). 4-Hydroxyisoleucine: A novel amino acid potentiator of insulin secretion. Diabetes.

[9] Shidfar F, Rahideh ST, Rajab A, Khandozi N, Hosseini S, Shidfar S, Mojab F (2014). The effect of sumac (Rhus coriaria L) powder on serum glycemic status, ApoB, ApoA-I and total antioxidant capacity in type 2 diabetic patients. Iran. J. Pharm. Res.

[10] Wong FC, Yong AL, Ting EP, Khoo SC, Ong HC, Chai TT (2014). Antioxidant, metal chelating, anti-glucosidase activities and phytochemical analysis of selected tropical medicinal plants. Iran. J. Pharm. Res.

[11] Browicz K, Zohary D (1996). The genus Amygdalus L(Rosaceae): Species relationships, distribution and evolution under domestication. Genet. Resour. Crop Ev.

[12] Sorkheh K, Shiran B, Khodambashi M, Rouhi V, Mosavei S, Sofo A (2012). Exogenous proline alleviates the effects of H2O2-induced oxidative stress in wild almond species. Russ. J. Plant Physiol.

[13] Parsa A (1960). Medicinal plants and drugs of plant origin in Iran. IV. Plant Foods Hum. Nutr. (Formerly Qualitas Plantarum).

[14] Babaei H, Sadeghpour O, Nahar L, Delazar A, Nazemiyeh H, Mansouri MR, Poursaeid N, Asnaashari S, Moghadam SB, Sarker SD (2008). Antioxidant and vasorelaxant activities of flavonoids from Amygdalus lycioides var horrida. Turkish J. Biol.

[15] Tiwari P, Kumar B, Kaur M, Kaur G, Kaur H (2011). Phytochemical screening and Extraction: A Review. Internationale Pharmaceutica Sciencia.

[16] El Far MM, Taie HA (2009). Antioxidant activities, total anthocyanins, phenolics and flavonoids contents of some sweetpotato genotypes under stress of different concentrations of sucrose and sorbitol. Aust. J. Basic Appl. Sci.

[17] Cheng YZ, Lee WJ, Chen LJ, Cheng JT, Chen MF (2012). Increase of myocardial performance by Rhodiola–ethanol extract in diabetic rats. J. Ethnopharmacol.

[18] Scherle W (1970). A simple method for volumetry of organs in quantitative stereology. Mikroskopie.

[19] Muhlfeld C, Nyengaard JR, Mayhew TM (2010). A review of state-of-the-art stereology for better quantitative 3D morphology in cardiac research. Cardiovasc. Pathol.

[20] Nyengaard JR (1999). Stereologic methods and their application in kidney research. J. Am. Soc. Nephrol.

[21] Bangle R Jr (1956). Factors influencing the staining of beta-cell granules in pancreatic islets with various basic dyes, including paraldehyde-fuchsin. Am. J. Pathol.

[22] Daisy P, Jasmine R, Ignacimuthu S, Murugan E (2009). A novel steroid from Elephantopus scaber L an ethnomedicinal plant with antidiabetic activity. Phytomedicine.

[23] Yanardag R, Ozsoy-Sacan O, Bolkent S, Orak H, Karabulut-Bulan O (2005). Protective effects of metformin treatment on the liver injury of streptozotocin-diabetic rats. Hum. Exp. Toxicol.

[24] Cam MC, Li WM, McNeill JH (1997). Partial preservation of pancreatic beta-cells by vanadium: Evidence for long-term amelioration of diabetes. Metabolism.

[25] Zini E, Osto M, Franchini M, Guscetti F, Donath MY, Perren A, Heller RS, Linscheid P, Bouwman M, Ackermann M, Lutz TA, Reusch CE (2009). Hyperglycaemia but not hyperlipidaemia causes beta cell dysfunction and beta cell loss in the domestic cat. Diabetologia.

[26] Kannur D, Hukkeri V, Akki K (2006). Antidiabetic activity of Caesalpinia bonducella seed extracts in rats. Fitoterapia.

[27] Lebovitz HE, Feinglos MN (1983). Mechanism of action of the second-generation sulfonylurea glipizide. Am. J. Med.

[28] Nuraliev I, Avezov G (1991). The efficacy of quercetin in alloxan diabetes. Eksp. Klin. Farmakol.

[29] Gattazzo F, Urciuolo A, Bonaldo P (2014). Extracellular matrix: A dynamic microenvironment for stem cell niche. Biochim. Biophys. Acta.

[30] Wellen KE, Hotamisligil GS (2003). Obesity-induced inflammatory changes in adipose tissue. J. Clin. Invest.

[31] Pickup JC (2004). Inflammation and activated innate immunity in the pathogenesis of type 2 diabetes. Diabetes Care.

[32] Rashid K, Sil PC (2015). Curcumin enhances recovery of pancreatic islets from cellular stress induced inflammation and apoptosis in diabetic rats. Toxicol. Appl. Pharmacol.

[33] Choudhary NK, Sharma S, Jha AK, Karchuli MS, Dwivedi J (2012). Antioxidant potential and protection of pancreatic beta- cells by Calotropis gigantea in streptozocin induced diabetic rats. J. Complement. Integr. Med.

[34] Gaggeri R, Rossi D, Christodoulou MS, Passarella D, Leoni F, Azzolina O, Collina S (2012). Chiral flavanones from Amygdalus lycioides Spach: Structural elucidation and identification of TNFalpha inhibitors by bioactivity-guided fractionation. Molecules.

[35] Salahuddin M, Jalalpure SS (2010). Antidiabetic activity of aqueous fruit extract of Cucumis trigonus Roxb in streptozotocin-induced-diabetic rats. J. Ethnopharmacol.

[36] Reasner CA (2005). What is the most effective strategy for managing diabetic dyslipidaemia?. Atheroscler. Suppl.

[37] Zhang S, Xu H, Yu X, Wang Y, Sun F, Sui D (2015). Simvastatin ameliorates low-dose streptozotocin-induced type 2 diabetic nephropathy in an experimental rat model. Int. J. Clin. Exp. Med.

[38] Al-Malki AL, El Rabey HA (2015). The antidiabetic effect of low doses of Moringa oleifera Lam seeds on streptozotocin induced diabetes and diabetic nephropathy in male rats. Biomed. Res. Int.

